# Self-Regulated Particle Swarm Multi-Task Optimization

**DOI:** 10.3390/s21227499

**Published:** 2021-11-11

**Authors:** Xiaolong Zheng, Deyun Zhou, Na Li, Tao Wu, Yu Lei, Jiao Shi

**Affiliations:** School of Electronics and Information, Northwestern Polytechnical University, 127 West Youyi Road, Xi’an 710072, China; xlzheng@mail.nwpu.edu.cn (X.Z.); dyzhounpu@nwpu.edu.cn (D.Z.); linaflydream@mail.nwpu.edu.cn (N.L.); tao_woe@mail.nwpu.edu.cn (T.W.); jiaoshi@nwpu.edu.cn (J.S.)

**Keywords:** evolutionary multitasking, evolutionary multi-task optimization, multi-task optimization, knowledge transfer, particle swarm optimization

## Abstract

Population based search techniques have been developed and applied to wide applications for their good performance, such as the optimization of the unmanned aerial vehicle (UAV) path planning problems. However, the search for optimal solutions for an optimization problem is usually expensive. For example, the UAV problem is a large-scale optimization problem with many constraints, which makes it hard to get exact solutions. Especially, it will be time-consuming when multiple UAV problems are waiting to be optimized at the same time. Evolutionary multi-task optimization (EMTO) studies the problem of utilizing the population-based characteristics of evolutionary computation techniques to optimize multiple optimization problems simultaneously, for the purpose of further improving the overall performance of resolving all these problems. EMTO has great potential in solving real-world problems more efficiently. Therefore, in this paper, we develop a novel EMTO algorithm using a classical PSO algorithm, in which the developed knowledge transfer strategy achieves knowledge transfer between task by synthesizing the transferred knowledges from a selected set of component tasks during the updating of the velocities of population. Two knowledge transfer strategies are developed along with two versions of the proposed algorithm. The proposed algorithm is compared with the multifactorial PSO algorithm, the SREMTO algorithm, the popular multifactorial evolutionary algorithm and a classical PSO algorithm on nine popular single-objective MTO problems and six five-task MTO problems, which demonstrates its superiority.

## 1. Introduction

Multi-task optimization (MTO) [1,2,3] studies how to effectively and efficiently optimize multiple optimization problems simultaneously, and has been developed to be a new research area in the field of optimization. Suppose there are *k* optimization tasks to be optimized at the same time, each task to be a minimization problem. The mathematic description for an MTO problem can be: $\left\{x_{1}^{*},x_{2}^{*},\ldots ,x_{k}^{*}\right\}=argmin\left\{f_{1}\left(x_{1}\right),f_{2}\left(x_{2}\right),\ldots ,f_{k}\left(x_{k}\right)\right\}$, in which the candidate solution $x_{j}$ and the global optimal solution $x_{j}^{*}$ are both in a $D_{j}$-dimensional search space $X_{j},j=1,2,\ldots ,k$. Function $f_{j}$ with $f_{j}:X_{j}\to ℜ$, is the objective function of the *j*-th task $T_{j}$. By implicitly leveraging upon the underlying synergies between these *k* objection function landscapes, MTO can enable accelerated convergence towards the global optima of all these tasks simultaneously [4,5,6,7]. Therefore, MTO is very promising in improving the optimization performance of multiple real-world problems, such as the path planning for multiple UAVs. Note that, UAV path planning problem aims at finding a satisfactory path within moderate computation resources and affordable time [8].

To solve the above MTO problems efficiently, many optimizers from the field of optimization can be employed to develop different MTO solvers. For example, Bayesian optimization is developed to handle these MTO problems efficiently in [1]. However, the newly emerged evolutionary MTO (EMTO) [2,3] has been more popular over the years [9,10,11]. EMTO utilizes the population-based evolutionary computation techniques (ECs) to optimize an MTO problem. EMTO tries to exploit the population of ECs to realize implicit knowledge transfer across different optimization tasks during the optimization of an MTO problem.Because the candidate solutions of the population have already implicitly carried the knowledge or information of a task during the task’s optimization process.

Over the years, many EMTO algorithms have been proposed. In [2], Gupta et al. utilized the designed population’s cultural parts to affect the genetic operations of the employed classical genetic algorithm [12], so as to propose the efficient multifactorial evolutionary algorithm (MFEA). As a kind of EMTO algorithms based on genetic algorithm, MFEA has inspired many researchers to develop a lot of efficient EMTO solvers [13,14,15]. For example, the proposed multifactorial evolutionary algorithm with resource reallocation (MFEARR) in [16], reset the parameter rmp of knowledge transfer in MFEA via the survival rate of divergents, for the purpose of suppressing ineffective cross-task knowledge transfer. Divergents are the offspring generated by two parents who are excellent at different tasks. Among the rest solvers, we put our attention to the self-regulated knowledge transfer scheme in [3]. In this scheme, task relatedness is local and dynamic as it is explored and captured via the evolving population. Then, knowledge transfer adapts to this dynamic task relatedness via the re-creation of task groups for all component tasks, and via the evaluation of population on corresponding selected set of tasks. Besides the above solvers that based on genetic algorithm, many researchers put their effect on EMTO using particle swarm optimization(PSO) [17]. In [18], Feng et al. developed the bio-cultural models in MFEA into a stander PSO solver to realize a novel multifactorial PSO (MFPSO) algorithm. In [19], Cheng et al. optimized each of the component tasks using one unique population (i.e., swarm), and achieved inter-task knowledge transfer by replacing the currently global best optima of a task with that of a different task when updating population’s velocity, so as to propose a multitasking coevolutionary PSO (MT-CPSO) algorithm. In [20], Song et al. extended a popular dynamic multi-swarm optimization (i.e., DMS-PSO) algorithm into EMTO and proposed a multitasking multi-swarm optimization (MTMSO) algorithm. In MTMSO, each population is responsible for one unique task, and knowledge transfer across these population is implemented via the probabilistic crossover on these population’s personal bests. In addition, there are many other EMTO solvers proposed in recent years [21,22,23,24,25,26,27].

Considering the above self-regulated knowledge transfer scheme, a developed self-regulated evolutionary multi-task optimization (SREMTO) algorithm has demonstrated its superiority on the test suites as presented in the paper [3]. However, only works based on genetic algorithms are being studied. The effectiveness and efficiency of the scheme on PSO is still yet to be explored. Moreover, PSO has been one of the most popular optimization techniques for its simplicity and rapid convergence rate in the evolutionary computation community in the past few decades [28,29,30]. Therefore, the incorporation of the scheme with the PSO is very promising.

Therefore, this paper tries to propose a more efficient EMTO solver by incorporating the self-regulated knowledge transfer scheme with a classical PSO algorithm and redesigning knowledge transfer strategy. Befittingly, the proposed algorithm is referred to as a self-regulated particle swarm multi-task optimization(SRPSMTO) algorithm. Note that, two novel knowledge transfer strategies are developed along with two versions of the proposed SRPSMTO. According to the paper [3], task relatedness in this scheme is explored and captured by quantifying the abilities (i.e., ability vectors) of every individual on solving different component tasks. In contrast to the scheme to recreate task groups using population’s ability vectors during each generation, this paper tries to introduce a newly devised knowledge transfer strategy to better fit into the classical evolution structure of a PSO algorithm and the dynamic relatedness between tasks. In the strategy, the task’s impact on an individual is adapted to the individual’s historical performance on the task via the knowledge integration operation and the probability selection operation, where the knowledge integration operation synthesizes the impacts from a set of tasks that are selected by the probability selection operation. Two versions of knowledge transfer strategies are developed along with two versions of the proposed algorithm, for the purpose of studying the performance difference of the proposed algorithm when using these two strategies. After updating the population’s velocities and positions, each individual of the population is evaluated on a selected set of tasks that are selected using probability corresponding to the individual’s ability on these tasks. Several experiments are conducted on a bi-task MTO problem set and a 5-task MTO problem set, which demonstrated the superiority of the proposed SRPSMTO.

The remaining paper will be organized as follows. The related background of EMTO, the self-regulated knowledge transfer scheme and particle swarm optimization are reviewed in Section 2. In Section 3, the motivation of this paper is firstly shown, which is then followed by the details of the proposed algorithm. Several experiments are conducted in Section 4, the results of which highlights the performance of the proposed SRPSMTO. Finally in Section 5, some important directions are revealed for future research.

## 2. Background

### 2.1. Evolutionary Multi-Task Optimization

Over the past few decades, population-based evolutionary computation techniques (ECs) have been established, and have shown promising results in handling nonlinear, multimodal and NP-hard problems [31,32,33,34]. However most of these techniques can only solve a single optimization problem. The implicit parallelism of population has not been fully explored and exploited. As aforementioned, MTO can study the optimization problems via the simultaneous optimization of multiple tasks, under an assumption that the information of one task may be helpful in improving the optimization efficiency of the other tasks during optimization. The population-based characteristic of ECs makes themselves suitable for solving these MTO problems. Hence, evolutionary multi-task optimization (EMTO) [2] is developed as an efficient framework to deal with the MTO problems [4,5,10,21].

**Figure 1:**
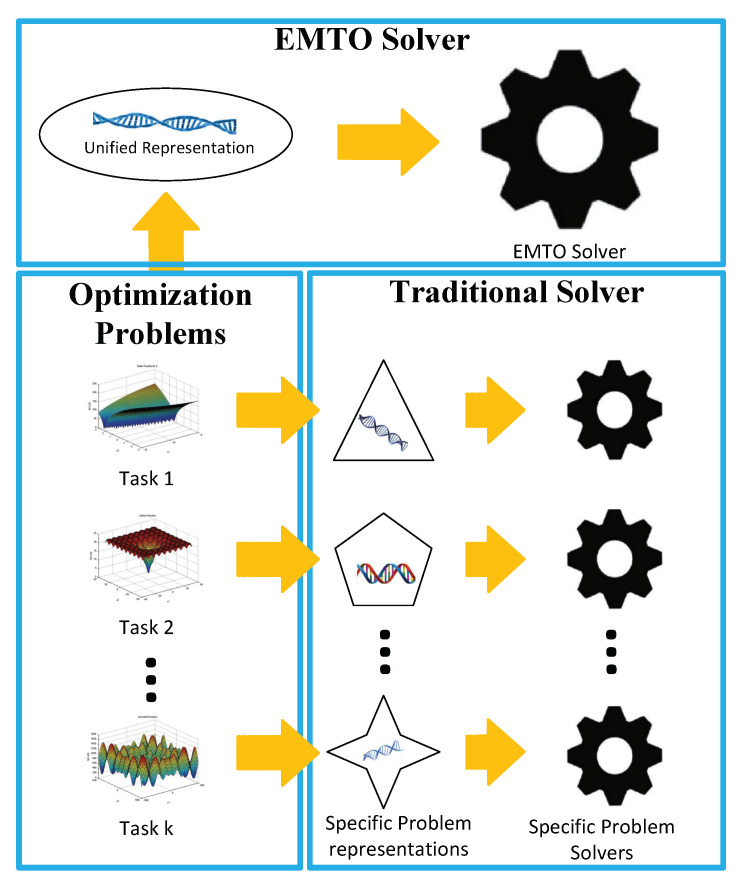
Highlighting the distinction between EMTO and traditional evolutionary optimization. In EMTO, all task-specific search spaces will be encoded within a unified representation space. An EMTO solver will simultaneously optimize all involved problems within this unified space, and output the found best solutions for all problems. Contrarily, traditional optimization independently optimizes each problem in a problem-specific search space.

EMTO is totally different from traditional evolutionary optimization. In traditional optimization, each solver independently optimizes an optimization problem within a problem-specific search spaces. However, EMTO simultaneously solves these tasks in a unified representation space [2] using only one EMTO solver as illustrated in Figure 1. A popular unified representation in EMTO community is a random key approach [35], which encoded each decision variable of problem-specific search spaces via a random key between 0 and 1. By using such a unified representation, the knowledge or useful information from one of all involved tasks can be implicitly utilized by the rest tasks, avoiding the problem of complex knowledge representation.

To efficiently solve MTO problems, the multifactorial evolutionary algorithm (MFEA) [2] is proposed as the first EMTO solver and has inspired many researchers. MFEA takes inspiration from the bio-cultural models of multifactorial inheritance, which believes that the complex developmental traits among offspring come from the interactions of genetic and cultural factors. In particular, cultural effects are incorporated via two aspects of the models in MFEA, that is (1) assortative mating, which considers that individuals prefer to mate with those belonging to the same cultural background; and (2) vertical cultural transmission, which believes that the phenotype of offspring is affected by the phenotype of its parents directly. Accordingly, MFEA splits the population into different skill groups, each representing a kind of culture. Subsequently, MFEA transfers the genetic materials between tasks in an implicit method, i.e., (1) a random probability (rmp) which allows individuals from different tasks to mate freely and (2) selective imitation in which the generated offspring can imitate the skill factor of either of its parent. Ultimately, the implemented MFEA achieves promising results in simultaneously optimizing multiple tasks.

### 2.2. Self-Regulated Knowledge Transfer Scheme

An MTO problem usually consists of multiple component tasks. Intuitively, for these tasks, the higher tasks relatedness, the more common knowledge or re-useful information. As each of these component tasks has a different fitness landscape, different pairs of component tasks may probably possess different tasks relatedness. Consequently, one component task may probably benefit from (or assist in) each of the rest component tasks to different extents. Hence, a self-regulated knowledge transfer scheme [3] was proposed by dynamically capturing tasks relatedness and accordingly adjusting the degree of knowledge transfer across these tasks during their optimization processes, so as to further improve the efficiency of knowledge transfer.

To capture tasks relatedness for an MTO problem during multitasking, the scheme refers to a popular used method [36], which computes task relatedness as Spearman’s rank correlation coefficient [36] for each task pair after randomly sampling tasks’ solution spaces and obtaining the factorial costs and the factorial ranks of these samples. The factorial costs and the factorial ranks defined in [2] is given in Definitions 1 and 2. Therefore, for different task pairs, the more similar in samples’ ranks, the higher correlation between task pairs. Based on this observation, the scheme defines an ability vector (Definition 3) to quantify the ability of *n*-sized population ($pop={\left\{p_{i}\right\}}_{i=1}^{n}$) on each of the component tasks. As a result, for two component tasks, the higher tasks relatedness between them, the more similar in the population’s ranks (i.e., factorial ranks), and the more similar in the population’s abilities (i.e., ability vectors). Accordingly, the scheme recreates task groups for each component task based on the ability vectors of the evolving population at each generation, and evaluates the offspring’s quality of these task groups on some randomly selected tasks with selection probabilities being the element values in their ability vectors. Consequently, the more tasks relatedness, the more common groups members with respect to these tasks, and therefore the higher degree of knowledge transfer between these tasks.

**Definition** **1.** *(Factorial Cost): Considering a given task $T_{j}$, $\Psi_{i,j}=\lambda \cdot \delta_{i,j}+f_{i,j}$ is defined as the factorial cost of individual $p_{i}$; in which λ is a large penalizing multiplier, and the objective value $f_{i,j}$ and the total constraint violation $\delta_{i,j}$ of $p_{i}$ are with respect to $T_{j}$. Notably, if $p_{i}$ is feasible with respect to $T_{j}$, then we have $\Psi_{i,j}=f_{i,j}$.*

**Definition** **2.** *(Factorial Rank): The factorial rank $rank_{i,j}$ of an individual $p_{i}$ on task $T_{j}$ is defined as the index of $p_{i}$ in the list of population members sorted in ascending order with respect to its factorial cost $\Psi_{i,j}$.*

**Definition** **3.** *(Ability Vector): Individual $p_{i}$’s ability vector is denoted as $\phi_{i}={\left\{\phi_{i,j}\right\}}_{j=1}^{k}$. $\phi_{i,j}$ indicates $p_{i}$’s ability on handling component task $T_{j}$, and is defined as $\phi_{i,j}=f_{m}\left(rank_{i,j}\right)$ where $rank_{i,j}$ is $p_{i}$’s factorial rank on $T_{j}$ and $f_{m}$ monotonically and decreasingly maps the factorial rank from the range of [1, rmax] to the range of [0.0,1.0]. The term rmax is the maximal value of the rank and typically equals to population size n.*

In [3], the function $f_{m}$ in Definition 3 is defined as:

$$\phi_{i,j}=f_{m}\left(rank_{i,j}\right)=\left\{\begin{array}{c} a_{1}\cdot rank_{i,j}+b_{1},rank_{i,j}\in \left[1,m\right].\\ a_{2}\cdot rank_{i,j}+b_{2},rank_{i,j}\in \left[m+1,n\right]. \end{array}\right.$$

in which *n* is population size and *m* (set to n/k when there is *k* component tasks) indicates the size of each of the tasks groups. As a line can be represented by two points, parameters $a_{1}$ and $b_{1}$ are determined via points (1, 1.0) and (*m*, TH) and parameters $a_{2}$ and $b_{2}$ are determined via points (m+1, TH) and (*n*, 0.0). (1, 1.0), (*m*, TH) and (*n*, 0.0) are the end points of these line segments. Parameter TH can control the slopes of these two line segments via manual setting in the range of [0.0, 1.0]. With such kind of definition, superior individuals for each task can have more chance to be selected into the corresponding task group while inferior individuals still have room for being selected.

The self-regulated knowledge transfer scheme was implemented into a self-regulated evolutionary multi-task optimization (SREMTO) algorithm [3]. After comparing with several efficient EMTO algorithms on two MTO test suites, the SREMTO as well as the scheme, has demonstrated its superiority.

### 2.3. Particle Swarm Optimization

Particle swarm optimization (PSO) [17] is a kind of intelligence algorithm that simulates the predation behavior of birds, in which social information are shared between these birds. Each of the birds, called individuals (i.e., candidate solution for optimization problem), can benefit from its own experience and all other companions’ previous experience during its food search process.

In PSO, an individual is represented by a position vector $x_{i}=\left(x_{i,1},x_{i,2},\ldots ,x_{i,D}\right),i=1,2,\ldots ,n$ and a velocity vector $v_{i}=\left(v_{i,1},v_{i,2},\ldots ,v_{i,D}\right),i=1,2,\ldots ,n$, where *n* is population size and *D* is the dimensionality of the search space of a given optimization problem. Velocity vector $v_{i}$ indicates an individual $p_{i}$’s distance and direction in the next step of movement. An individual’s movement in PSO is influenced by three components, i.e., the inertia or momentum component, the cognitive component and the social component. The inertia component describes the ability of an individual to keep track of its previous flow direction. The cognitive component identifies the tendency of an individual $p_{i}$ to move back to its personal best position found by itself so far, labeled as ${\mathrm{pbest}}_{i}$. The social component accounts for the influence of the so far found best position by the whole population, which is labeled as gbest. Formally, the updates of an individual $p_{i}$’s velocity and position on *d*-th dimension from generation *g* to g+1 are as defined as follows:

(1) $$v_{i,d}^{g+1}=wv_{i,d}^{g}+c_{1}r_{1}\left(pbest_{i,d}-x_{i,d}^{g}\right)+c_{2}r_{2}\left(gbest_{d}-x_{i,d}^{g}\right)$$

(2) $$x_{i,d}^{g+1}=x_{i,d}^{g}+v_{i,d}^{g+1}$$

where the parameter *w* is an inertia weight that adjusts the influence from the inertia component. $c_{1}$ and $c_{2}$ adjust the influence of the personal best ${\mathrm{pbest}}_{i}$ and the gbest respectively, and usually are real-valued in range [0,4]. Parameter $r_{1}$, $r_{2}$ are random numbers generated from a uniform distribution in range [0,1].

## 3. The Proposed Method

### 3.1. Motivations

As aforementioned, the self-regulated knowledge transfer scheme adapts knowledge transfer to task relatedness by dynamically exploring and capturing task relatedness during optimization. The relatedness of component tasks is important in influencing the optimization efficiency of EMTO. For the component tasks possessing high task relatedness, an EMTO solver can efficiently solve them all under the effects of knowledge transfer. On the contrary, for the component tasks with low task relatedness, the knowledge transfer may be useless, even harmful, to task optimization process. Therefore, the adaptation of knowledge transfer to task relatedness really matters in EMTO, and this self-regulated knowledge transfer scheme is one unique method to realize the adaptation.

Meanwhile, PSO has attained remarkable attention from researchers over the past decade for its ease of implementation and high efficiency in solving single objective problems, especially in optimizing continuous problems [37,38,39,40]. On the one hand, there are mainly two operations in a classical PSO algorithm as described above, i.e., velocity updating and position updating, and there are only three parameters that need to be adjusted, including the inertia weight *w*, the learning factor $c_{1}$ and $c_{2}$. Hence, PSO can be easily implemented. On the other hand, many versions of PSO have been proposed, and have been tested on many complicated problems which have demonstrated promising outcomes [41,42,43,44].

Therefore, the incorporation of the scheme with an classical PSO algorithm is very promising in generating an efficient EMTO solver. The details of the proposed solver can be seen at the next chapter.

### 3.2. Self-Regulated Particle Swarm MTO (SRPSMTO) Algorithm

PSO is a population-based search technique, in which individuals will share experience with each other, so that all of them can move towards better positions. To handle MTO problems effectively, the self-regulated knowledge transfer scheme [3] is incorporated with the PSO algorithm to develop an self-regulated particle swarm MTO (i.e., SRPSMTO) algorithm. To further obtain better performance, an effective inter-task knowledge transfer strategy is developed, in which task’s impact on an individual is adapted to the individual’s historical performance on the task via the knowledge integration operation and the probability selection operation. Two versions of the inter-task knowledge transfer strategies are developed, and each version is corresponding to a version of the SRPSMTO algorithm. The basic structure of the proposed algorithm is shown in Algorithm 1.

In SRPSMTO, the randomly-initialized population are evenly separated into *k* subgroups at the beginning, each subgroup corresponding to a unique task. In the first generation, the individuals of a task’s subgroup are evaluated only on the task, to obtain their initial fitness, and their fitness on the other tasks are set to inf. After all individuals are evaluated, the SRPSMTO updates the ${\mathrm{pbest}}_{i},i=1,\ldots ,n$ and ${\mathrm{gbest}}_{j},j=1,\ldots ,k$ of population. Then the algorithm obtains individuals’ factorial ranks on all component tasks, and accordingly computes the individuals’ ability vectors according to the Definition 3. In step 5 and 6, the algorithm updates the velocities and positions of all individuals. After individuals move to new positions, the algorithm evaluates all individuals once again. But differing from the first generation, the individuals are now evaluated on a set of tasks that are randomly selected with selection probabilities being the element values in the ability vectors of the individuals. In step 7 and 8, the ${\mathrm{pbest}}_{i},i=1,\ldots ,n$, ${\mathrm{gbest}}_{j},j=1,\ldots ,k$ and the ability vectors of the individuals are updated again. If the stopping conditions are not satisfied, the algorithm steps into another runs again. Details of the proposed SRPSMTO will be discussed below.
**Algorithm 1** SRPSMTO.**Input:**    *n* (population size)    *k* (number of tasks)    $w,c_{1},c_{2}$ (PSO parameters)**Output:**   $\left\{x_{1}^{*},x_{2}^{*},\ldots ,x_{k}^{*}\right\}$ (the best solution achieved on each of the *k* component tasks)1:    Randomly generate a population pop of size *n*.2:   Evenly separate pop into *k* subgroups and evaluate each subgroup on one corresponding task.3:   Update the ${\mathrm{pbest}}_{i},i=1,\ldots ,n$ and ${\mathrm{gbest}}_{j},j=1,\ldots ,k$, and the ability vector $\phi_{i}={\left\{\phi_{i,j}\right\}}_{j=1}^{k}$ of individual $p_{i}$ in pop.4:   **while** (stopping conditions are not satisfied) **do**5:     Update positions (see Equation (2)).6:     Update velocities (see Algorithm 2 and 3).7:     Evaluate all individuals and Update the ${\mathrm{pbest}}_{i},i=1,\ldots ,n$ and ${\mathrm{gbest}}_{j},j=1,\ldots ,k$ (see Algorithm 4).8:      Update the ability vector of every individual.9:  **end while**

#### 3.2.1. Inter-Task Knowledge Transfer

In a canonical PSO, population move from place to place under the effects of three parts: the inertial component, the cognition component and the social component. The social component represents the impact from the so far found best position of the optimization problem. In EMTO, two or more component tasks are simultaneously optimized in one unified representation space. Therefore, each individual of the population will be influenced by multiple component tasks at a same time. With the incorporated SREMTO scheme, in this paper, the impact of these tasks on an individual can adapt to the individual’s historical performance on these tasks. In the following, an knowledge integration operation and a probability selection operation are developed to achieve flexibly inter-task knowledge transfer.

In the SREMTO scheme, the abilities of every individual on handling different component tasks have been quantified via the defined ability vector. Therefore, this paper devises a knowledge integration operation to integrate the impacts from different tasks, to assist an individual to move towards more promising regions in the near future. In this knowledge integration, the impacts of tasks’ so far found best positions are weighted and summed over by individual’s ability vector when the individual’s velocity is updated. As a result, different tasks can impact the individual’s movements to different extents, the degree of which is decided by the individual’s ability values on these tasks. If an individual can perform well on multiple tasks (i.e., possessing high ability vales on multiple tasks), the knowledge integration operation can help the individual move faster towards promising regions.

Intuitively, a task may probably have positive influence on an individual if the individual has good performance on the task in history. Therefore, a probability selection operation is devised to reduce the negative impacts from some tasks to a specific individual. Specifically, the movement of an individual is now impacted by a set of tasks, and each task is selected using individual’s ability value on the task as probability. As a result, each individual of population can have more chance to move towards better positions

In Algorithm 2, the knowledge integration operation and the probability selection operation are performed to update the velocity of every individual. In step 4, each task is selected with a probability of $\phi_{i,j}$, and is utilized to update an individual’s velocity. Once a task $T_{j}$ is selected, then the ${\mathrm{gbest}}_{j}$ of the task is employed to update the individual’s velocity via a weighted summation process as shown in step 5 and step 7.
**Algorithm 2** VelocityUpdate_V1.1:**for** individual $p_{i},i=1$ to *n* **do**2:   $temp_{d}=0$.3:   **for** task $T_{j},j=1$ to *k* **do**4:     **if** $rand<\phi_{i,j}$ **then**5:        $temp_{d}=temp_{d}+\phi_{i,j}\left(gbest_{j,d}-x_{i,d}^{g}\right)$.6:     **end if**7:     $v_{i,d}^{g+1}=wv_{i,d}^{g}+c_{1}r_{1}\left(pbest_{i,d}-x_{i,d}^{g}\right)+c_{2}r_{2}\left(temp_{d}\right)$.8:   **end for**9:**end for**

In Algorithm 3, we introduce another type of knowledge integration operation and the probability selection operation, to develop a second knowledge transfer strategy for the proposed algorithm. In the knowledge integration operation, the impacts of tasks’ so far found best positions from different tasks are weighted and summed over by individual’s ability vector as well. Meanwhile, a normalization process is devised to normalize this weighted sum as shown in step 8 and 10. In the probability selection operation, the task that an individual has the greatest ability value is ensured to be selected while the rest tasks are selected using probabilities as shown in step 2 and 5. These modifications enhance individuals’ optimization on the tasks that the individual performs well in history. Therefore, comparing to the Algorithm 2, the knowledge transfer strategy in Algorithm 3 may probably benefit the proposed algorithm when optimizing MTO problems consisting of more than two component tasks. Contrarily, Algorithm 3 may have less efficiency in knowledge transfer due to the limitation in the flexibility of knowledge transfer, especially when two optimized tasks are highly related.
**Algorithm 3** VelocityUpdate_V2.1:**for** individual $p_{i},i=1$ to *n* **do**2:   Get the index $j_{b}$ of the biggest in $\phi_{i}={\left\{\phi_{i,j}\right\}}_{j=1}^{k}$.3:   $temp_{d}=0,norm_{af}=0$.4:   **for** task $T_{j},j=1$ to *k* **do**5:     **if** $j=j_{b}$ or $rand<\phi_{i,j}$ **then**6:        $r_{2}=rand$7:        $temp_{d}=temp_{d}+r_{2}*\phi_{i,j}*\left(gbest_{j,d}-x_{i,d}^{g}\right)$.8:        $norm_{af}=norm_{af}+\phi_{i,j}$.9:     **end if**10:     $v_{i,d}^{g+1}=wv_{i,d}^{g}+c_{1}r_{1}\left(pbest_{i,d}-x_{i,d}^{g}\right)+c_{2}\left(temp_{d}/norm_{af}\right)$.11:   **end for**12:**end for**

#### 3.2.2. Selective Evaluation

The evaluation of population in a multitasking environment has to take more factors into consideration, because individuals are affected by multiple tasks simultaneously. In generally, we should evaluate an individual on all component tasks for better understanding the individual’s performance on all tasks, which, however, is computationally expensive. Therefore, an individual is evaluated on tasks that are selected with selection probabilities being the element values in its ability vectors as shown in step 4 in Algorithm 4. For these selected tasks, the task that an individual is most excellent at, is ensured to be selected. The rest of the tasks are selected with probabilities, and an individual’s fitness on tasks that has not been evaluated will be set to *∞* as given in step 7 of the Algorithm 4.
**Algorithm 4** Population Evaluation.1:**for** individual $p_{i},i=1$ to *n* **do**2:   Get the index $j_{b}$ of the biggest in $\phi_{i}={\left\{\phi_{i,j}\right\}}_{j=1}^{k}$.3:   **for** task $T_{j},j=1$ to *k* **do**4:     **if** $j\in j_{b}$ or $rand<\phi_{i,j}$ **then**5:        $fitness_{i,j}=f_{j}\left(position_{i}\right)$.6:     **else**7:        $fitness_{i,j}=\infty$.8:     **end if**9:     **if** $fitness_{i,j}$ is better than ${\mathrm{pbest}}_{i}$’s fitness on *j* **then**10:        Update the ${\mathrm{pbest}}_{i}$.11:        **if** $fitness_{i,j}$ is better than fitness of ${\mathrm{gbest}}_{j}$ **then**12:          Update the ${\mathrm{gbest}}_{j}$.13:        **end if**14:     **end if**15:   **end for**16:**end for**

After evaluation, an individual’s personal best (i.e., ${\mathrm{pbest}}_{i}$) is updated if its current fitness on any component tasks is better as shown in step 9 and step 10 of Algorithm 4, and the ${\left\{{\mathrm{gbest}}_{j}\right\}}_{j=1}^{k}$ are updated in step 11 and step 12 if the better ones are found.

## 4. Experiments

In this section, a set of experiments are conducted to evaluate the performance of the proposed SRPSMTO algorithm in comparison to the MFPSO [18], the SREMTO [3], the MFEA [2] and as well as a traditional PSO algorithm on two test suits. Meanwhile, an in-depth analysis is made to demonstrate the effectiveness and efficiency of the proposed SRPSMTO on handling MTO problems with 5 component tasks via the comparison with the PSO, the MFEA, the SREMTO. In the last experiment, a parameter study was made to study the impact of the population size in SRPSMTO. Before the experiments, we will first detail test problems and the experimental setup in the following.

### 4.1. Test Problems

Two suites of test problems are used in the following experiments. Test suite 1 contains nine MTO problems from the CEC 2017 Evolutionary Multi-Task Optimization Competition as shown in Table 1. Each of these problems are composed of two distinct single-objective optimization tasks which have their own global optima as well as their own dimension size and inter-task similarity (i.e., task relatedness). According to the overlapping degrees of their component tasks’ global optima, these problems can be classified into three categories, i.e., complete intersection (CI), partial intersection (PI) and no intersection (NI). The component tasks of the problems in CI set have the same global optima, and those in NI set will have totally different global optima. For PI set, the component tasks of each problem will have their own global optima which are only the same on some dimensions. Besides the above overlapping degrees, the nine MTO problems in test suite 1 can be classified into 3 classes if according to the inter-task similarity ($R_{s}$). That is, low similarity set when $R_{s}<0.2$, medium similarity set when $0.2\leq R_{s}\leq 0.8$ and high similarity set when $R_{s}>0.8$. Note that, $R_{s}$ measures the degree of inter-task similarity of different component tasks in an MTO problem, and is computed via the Spermans rank correlation coefficient [36]. $R_{s}$ is with value of range [−1, 1], and indicates the lowest and highest similarity of component tasks using its magnitude of range 0 and 1, respectively. Its positive and negative signs denote positive and negative similarity between component tasks, respectively.

Test suite 2 contains six five-task MTO problems, as shown in Table 2. Each of the six MTO problems contain five single-objective optimization tasks, and these tasks are constructed using a same basic function with the same space search ranges but different rotation and slight shift. All the five component tasks of each MTO problem are 50 dimensional, and have no intersection on their global optima. The details of the constructed MTO problems can be seen in the table.

Comparing to test suite 1, the MTO problems of test suite 2 are constructed with more than 2 component tasks. Hence, an EMTO solver’s performance on a more general case of multitasking can be tested.

To test the performance of the proposed algorithm, the following continuous objective functions are used to construct MTO problems by treating each of them as a task in test suites 1 and 2. These functions [45] are simply described as follow.

(1) Sphere function

$$f\left(x\right)=\sum_{i=1}^{D}x_{i}^{2}.$$

(2) Rosenbrock function

$$f\left(x\right)=\sum_{i=1}^{D-1}{[100\left(x_{i+1}-x_{i}^{2}\right)}^{2}+{\left(x_{i}-1\right)}^{2}].$$

(3) Perm0db function

$$f\left(x\right)=\sum_{i=1}^{D}{\left(\sum_{j=1}^{D}\left(j+\beta \right)\left(x_{j}^{i}-\frac{1}{j^{i}}\right)\right)}^{2},\beta =10.$$

(4) Levy function

$$\begin{array}{c} f\left(x\right)=\sin^{2}\left(\pi w_{1}\right)\\ +\sum_{i=1}^{D-1}{\left(w_{i}-1\right)}^{2}\left[1+10\sin^{2}\left(\pi w_{i}+1\right)\right]\\ +{\left(w_{d}-1\right)}^{2}\left[1+\sin^{2}\left(2\pi w_{d}\right)\right],\\ w_{i}=1+\frac{x_{i}-1}{4},i=1,\ldots ,D. \end{array}$$

(5) Ackley function

$$\begin{array}{c} f\left(x\right)=-20\exp\left(-0.2\sqrt{\frac{1}{D}\sum_{i=1}^{D}x_{i}^{2}}\right)-\\ \exp\left(\frac{1}{D}\sum_{i=1}^{D}\cos(2\pi x_{i})\right)+20+\exp\left(1\right). \end{array}$$

(6) Griewank function

$$f\left(x\right)=\sum_{i=1}^{D}\frac{x_{i}^{2}}{4000}-\prod_{i=1}^{D}\cos\left(\frac{x_{i}}{\sqrt{i}}\right)+1.$$

(7) Rastrigin function

$$f\left(x\right)=10D+\sum_{i=1}^{D}\left[x_{i}^{2}-10\cos\left(2\pi x_{i}\right)\right].$$

(8) Weierstrass function

$$\begin{array}{c} f\left(x\right)=\sum_{i=1}^{D}\left(\sum_{k=0}^{k\max}\left[a^{k}\cos\left(2\pi b^{k}\left(x_{i}+0.5\right)\right)\right]\right)\\ -D\sum_{k=0}^{k\max}\left[a^{k}\cos\left(2\pi b^{k}\cdot 0.5\right)\right],\\ a=0.5,b=3,k\max=20. \end{array}$$

(9) Schwefel function

$$f\left(x\right)=418.9829\times D-\sum_{i=1}^{D}x_{i}\sin\left({\left|x_{i}\right|}^{\frac{1}{2}}\right)$$

### 4.2. Experimental Setup

Four sets of experiments are conducted in the following to (1) demonstrate the effectiveness of knowledge transfer in the proposed SRPSMTO via the comparison with a traditional PSO algorithm using test suite 1; (2) compare the performance of the SRPSMTO with the MFPSO, the SREMTO and the popular MFEA using test suite 1; (3) evaluate the performance of the SRPSMTO on a more general case of multitasking using test suite 2 while in comparison to the PSO, the SREMTO and the MFEA; (4) study the impact of parameter settings (population size *n*) in the SRPSMTO using test suite 1. Note that, two versions of the proposed SRPSMTO are shown in these experiments. The SRPSMTO_V1 uses the strategy presented in Algorithm 2, and the SRPSMTO_V2 employs the strategy described in Algorithm 3 in the previous section.

The parameter settings for the MFPSO, the SREMTO and the MFEA are as suggested in the original literature. The settings for the PSO algorithm are according to Eberhart et al. [46], and are used in the proposed SRPSMTO. The details of parameter settings for these algorithm are summarized as follows:

1. Population size: n=50×k, *k* is the number of component tasks in an MTO problem [23]
2. Parameter settings in SRPSMTO:
  - inertia weight *w*: decreases linearly from 0.9 to 0.4
  - $c_{1}$ and $c_{2}$: $c_{1}=c_{2}=1.494$
  - ability vector: TH=0.13/k
3. Parameter settings in PSO are same as the settings in the SRPSMTO
4. Parameter settings in MFPSO:
  - random mating probability rmp: 0.3
  - inertia weight *w*: decreases linearly from 0.9 to 0.4
  - $c_{1}$, $c_{2}$ and $c_{3}$: 0.2
5. Parameter settings in SREMTO:
  - probability for crossover: $P_{\alpha }=0.7$
  - probability for mutation: $P_{\beta }=1.0$
  - distribution index of SBX: 1
  - distribution index of PM: 39
6. Parameter settings in MFEA:
  - random mating probability rmp: 0.3
  - distribution index of SBX: 2
  - distribution index of PM: 5

Each of the experiments is conducted for 30 runs. The maximum number of function evaluations (maxFEs) is used in all involved algorithms as the stopping criterion to terminate a run. The maxFEs is set to 100,000 ∗k in all experiments [36]. The best objective function error values (FEVs) are defined as the difference between the objective function value of the found solution and that of the global optimum. After the termination of a run, the mean and the standard deviation of the achieved FEVs over 30 runs are used to measure the performance of the algorithm in terms of optimization accuracy. To compare the performance of multiple algorithms in a multi-task scenario, a performance score as defined in [36] is employed. More specifically, assume that there are *Q* algorithms running on a *k*-task MTO test problem and each algorithm runs for *L* runs. The score of an algorithm *q* on this *k*-task MTO problem is obtained by $score_{q}=\sum_{j=1}^{k}\sum_{l=1}^{L}\left(I_{q,j,l}-\mu_{j}\right)/\sigma_{j}$, where $I_{q,j,l}$ is the achieved FEVs of algorithm *q* on the *j*-th task and *l*-th run, $\mu_{j}$ and $\sigma_{j}$ are respectively the mean and the standard deviation of the achieved FEVs with respect to the *j*-th task over all runs of all algorithms. For an algorithm, the smaller the score, the better the performance.

### 4.3. Experimental Results

#### 4.3.1. The Effectiveness of Knowledge Transfer in SRPSMTO

Two novel versions of knowledge transfer strategy are proposed in this paper, which are employed in the two versions of the proposed SRPSMTO respectively. To validate the effectiveness of knowledge transfer in the SRPSMTO, this experiment is conducted on the nine MTO problems from Table 1 in comparison with a traditional PSO algorithm. As the PSO employs same parameter settings as the SRPSMTO, the difference between them will demonstrate the effectiveness of knowledge transfer in the proposed SRPSMTO. Table 3 and Table 4 reports the means and bracketed standard deviations of the achieved FEVs over 30 runs as well as the scores for the algorithms.

Table 3 shows us that the SRPSMTO (i.e., SRPSMTO_V1) obtains better scores than the traditional PSO algorithm with a better mean score −2.88E+1 comparing to the 2.88E+1 of the PSO. Secifically, for all the nine MTO problems, SRPSMTO has demonstrated better results than the PSO. Especially on problem 1, 2, 5 and 7, the efficient knowledge transfer strategy in SRPSMTO brings with great improvement in optimization quality on at least one of the two component tasks. If we look at them in Table 1, we find that there are high similarities (i.e., $R_{s}$ ) between the component tasks in most of these problems, which then indicates the effectiveness of knowledge transfer in SRPSMTO as well as the promising future of multitasking.

In Table 4, the SRPSMTO (i.e., SRPSMTO_V2) obtains a mean score −2.38E+1, better than the mean score 2.38E+1 of the PSO. For all of the nine MTO problems, the SRPSMTO has demonstrated better performance than the PSO. Therefore, knowledge transfer in the proposed algorithm works efficiently.

**Figure 2:**
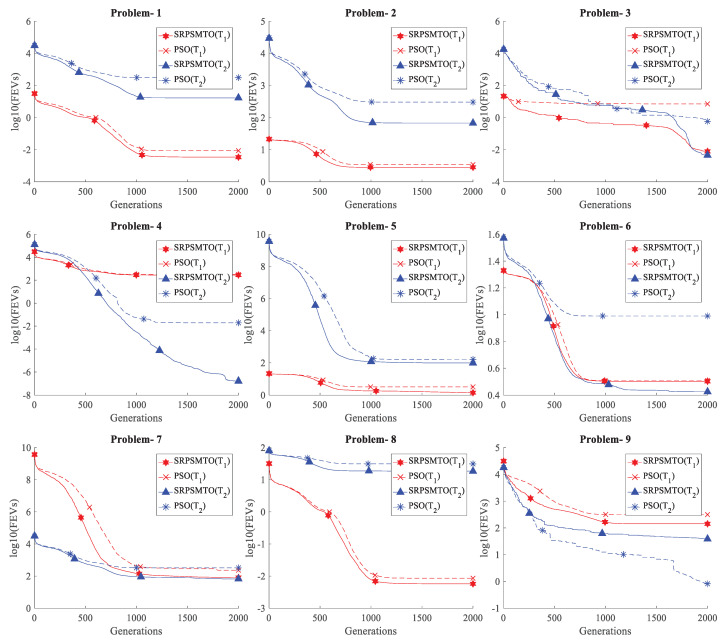
Averaged achieved FEVs convergence curves of the proposed SRPSMTO (i.e., SRPSMTO_V1) versus the PSO on the 9 test problems of test suite 1.

In Figure 2, the averaged achieved FEVs convergence curves of the proposed SRPSMTO (i.e., SRPSMTO_V1) are presented along with that of the PSO. The convergence curves of the SRPSMTO_V2 is similar to the SRPSMTO_V1’s, and therefore will not be presented here. From these figures, we can find that the benefits of multitasking can show up at the very beginning of optimization. In the early 500 generations, the convergence curves of most tasks of SRPSMTO have shown faster convergence trend than that of the PSO. Only on the task $T_{2}$ of problem 9 whose $R_{s}$ takes a value equal to 0, the SRPSMTO demonstrates slow convergence trend.

#### 4.3.2. Comparison of SRPSMTO with MFPSO, SREMTO and MFEA on Test Suite 1

To learn about the performance of the proposed SRPSMTO, this experiment compares the SRPSMTO with the MFPSO, the SREMTO and the popular MFEA from the field of multitasking. Both the two versions of SRPSMTO (i.e., SRPSMTO_V1 and SRPSMTO_V2) are considered. The experiment is conducted on the popularly used benchmark that is given in Table 1. Table 5 shows us the means and bracketed standard deviations of the achieved FEVs over 30 runs as well as the scores for all algorithms.

As shown in Table 5, the two versions of the SRPSMTO have obtained mean scores with value −4.21E+1 and −3.77E+1, respectively, which is better than that of the MFPSO (with a mean score 9.41E+1), the SREMTO (with a mean score −2.59E+1) and that of the MFEA (with a mean score 1.15E+1). In specific, the SRPSMTO_V1 achieved the best results on problem 1, 2, 3, 5 and 6, and the SRPSMTO_V2 achieved the best results on problem 9. The SREMTO achieved the best results on three of the nine problems, i.e., problem 4, 7 and 8, where both the two versions of the SRPSMTO can still achieve good enough results as shown in the table. Therefore, both the two version of the proposed SRPSMTO have demonstrated superiority on this test suite.

#### 4.3.3. Comparison of SRPSMTO with PSO, SREMTO and MFEA on Test Suite 2

To validate the efficiency of the proposed SRPSMTO on handling MTO problems with more than two-component tasks, we conduct this experiment on the six five-task MTO problems from test suite 2, as given in Table 2. The MFPSO is not suitable in handling these problems, and will not be considered here. Table 6 shows us the means and bracketed standard deviations of the achieved FEVs over 30 runs as well as the scores for all algorithms.

As shown in Table 6, both the two versions of the proposed SRPSMTO achieved better mean scores compared to the PSO, the SREMTO and the MFEA, with −4.44E+1 and −4.64E+1 comparing to the −2.34E+1 of PSO, the 5.43E+1 of SREMTO and the 5.98E+1 of MFEA. Especially for problems 1, 2 and 4, the SRPSMTO achieved the best results. Therefore, both versions of the proposed SRPSMTO have demonstrated their superiority on handling MTO problems with more than 2-component tasks.

#### 4.3.4. Parameters Analysis

This subsection studies the impact of population size on the proposed SRPSMTO (SRPSMTO_V1) using the nine MTO problems from test suite 1. The other parameter settings are kept the same as the settings in the previous experiments. Table 7 reports the means, the bracketed standard deviations and the scores of the achieved FEVs over 30 runs. The SRPSMTO_V2 performs similar results as the SRPSMTO_V1, hence, we only show SRPSMTO_V1’s results here.

The experimental results in Table 7 show us that, as the population size *n* increases from 20 to 180, the mean scores of the SRPSMTO under each parameter settings are getting smaller and smaller, which indicates that the performance of the proposed SRPSMTO generally gets better and better. When n=100, the algorithm outputs the best results (with a mean score −2.44E+1), which is also a popular setting in most of the EMTO solvers.

## 5. Concluding Remarks

In this paper, we proposed a SRPSMTO algorithm by incorporating the self-regulated knowledge transfer scheme with a classical PSO algorithm. Some empirical studies have been conducted on the proposed algorithm. The results show that the developed knowledge transfer strategies in SRPSMTO are efficient, and can achieve the algorithm with high performance on the two test suites comparing to the MFPSO, the SREMTO, the MFEA and the original classical PSO algorithm. Meanwhile, parameter analysis is conducted in the final part of the experiments to study the impact of population size on the algorithm’s performance.

As knowledge transfer plays an important role in an EMTO solver, in the future, more strategies can be explored to further improve the solver’s efficiency on multitasking. Particularly, many methods in machine learning can inspire us on designing more efficient knowledge transfer strategies. For example, transfer learning models [47] may be useful in helping knowledge transfer of multitasking. Additionally, to solve real world problems, such as permutation flow shop scheduling problems [48] and quadratic assignment problems [49], further studies may be required to find a method that updates the particles’ velocities.

## Figures and Tables

**Table 1:** Description of the nine MTO test problems in test suite 1.

Problem	Component Task	Degree of Intersection	*D*	$R_{s}$
1	$T_{1}$: Grewank	Complete intersection	50	1.00
	$T_{2}$: Rastrigin			
2	$T_{1}$: Ackely	Complete intersection	50	0.23
	$T_{2}$: Rastrigin			
3	$T_{1}$: Ackely	Complete intersection	50	0.00
	$T_{2}$: Schwefel			
4	$T_{1}$: Rastrigin	Partial intersection	50	0.87
	$T_{2}$: Sphere			
5	$T_{1}$: Ackely	Partial intersection	50	0.22
	$T_{2}$: Rosenbrock			
6	$T_{1}$: Ackely	Partial intersection	50($T_{2}$: 25)	0.07
	$T_{2}$: Weierstrass			
7	$T_{1}$: Rosenbrock	No intersection	50	0.94
	$T_{2}$: Rastrigin			
8	$T_{1}$: Griewank	No intersection	50	0.37
	$T_{2}$: Weierstrass			
9	$T_{1}$: Rastrigin	No intersection	50	0.00
	$T_{2}$: Weierstrass			

**Table 2:** Description of the six MTO problem sets in test suite 2.

Problem	Basic Function	Search Range	Degree of Intersection	*D*
1	Rosenbrock	[−50,50]	No intersection	50
2	Ackley	[−50,50]	No intersection	50
3	Rastrigin	[−50,50]	No intersection	50
4	Griewank	[−100,100]	No intersection	50
5	Weierstrass	[−0.5,0.5]	No intersection	50
6	Schwefel	[−500,500]	No intersection	50

**Table 3:** Comparison of SRPSMTO (SRPSMTO_V1) and PSO in terms of the means, bracketed standard deviations and scores of the best achieved FEVs over 30 runs on test suite 1. The better results are shown in bold.

Problem	Task	SRPSMTO_V1		PSO	
		Mean(Std)	Score	Mean(Std)	Score
1	$T_{1}$	3.45E−3(7.55E−3)	**−3.75E+1**	8.70E−3(7.41E−3)	3.75E+1
	$T_{2}$	1.69E+1(3.33E+1)		3.08E+2(7.88E+1)	
2	$T_{1}$	2.84E+0(6.62E−1)	**−3.78E+1**	3.44E+0(6.57E−1)	3.78E+1
	$T_{2}$	6.76E+1(2.67E+1)		3.04E+2(1.02E+2)	
3	$T_{1}$	1.01E−2(9.24E−3)	**−2.44E+1**	7.12E+0(1.01E+1)	2.44E+1
	$T_{2}$	7.15E−3(1.04E−2)		5.83E−1(1.05E+0)	
4	$T_{1}$	2.83E+2(7.26E+1)	**−1.02E+1**	3.13E+2(9.37E+1)	1.02E+1
	$T_{2}$	1.82E−7(4.40E−7)		1.94E−2(8.38E−2)	
5	$T_{1}$	1.44E+0(9.40E−1)	**−3.83E+1**	3.26E+0(6.39E−1)	3.83E+1
	$T_{2}$	9.79E+1(3.11E+1)		1.64E+2(6.83E+1)	
6	$T_{1}$	3.19E+0(8.22E−1)	**−2.70E+1**	3.21E+0(6.83E−1)	2.70E+1
	$T_{2}$	2.66E+0(8.16E−1)		9.77E+0(2.48E+0)	
7	$T_{1}$	8.36E+1(4.07E+1)	**−4.13E+1**	2.33E+2(1.66E+2)	4.13E+1
	$T_{2}$	6.69E+1(6.15E+1)		3.26E+2(9.38E+1)	
8	$T_{1}$	5.75E−3(8.13E−3)	**−2.99E+1**	8.57E−3(8.81E−3)	2.99E+1
	$T_{2}$	1.85E+1(3.31E+0)		3.11E+1(4.93E+0)	
9	$T_{1}$	1.42E+2(8.54E+1)	**−1.23E+1**	3.15E+2(1.04E+2)	1.23E+1
	$T_{2}$	4.07E+1(1.04E+2)		8.19E−1(1.73E+0)	
Mean		-	**−2.88E+1**	-	2.88E+1

**Table 4:** Comparison of SRPSMTO (SRPSMTO_V2) and PSO in terms of the means, bracketed standard deviations and scores of the best achieved FEVs over 30 runs on test suite 1. The better results are shown in bold.

Problem	Task	SRPSMTO_V2		PSO	
		Mean(Std)	Score	Mean(Std)	Score
1	$T_{1}$	6.17E−3(8.16E−3)	**−3.21E+1**	8.70E−3(7.41E−3)	3.21E+1
	$T_{2}$	2.86E+1(4.06E+1)		3.08E+2(7.88E+1)	
2	$T_{1}$	3.35E+0(7.81E−1)	**−2.59E+1**	3.44E+0(6.57E−1)	2.59E+1
	$T_{2}$	9.15E+1(4.48E+1)		3.04E+2(1.02E+2)	
3	$T_{1}$	1.29E−1(1.93E−1)	**−1.28E+1**	7.12E+0(1.01E+1)	1.28E+1
	$T_{2}$	6.20E−1(1.37E+0)		5.83E−1(1.05E+0)	
4	$T_{1}$	3.03E+2(9.57E+1)	**−6.41E+0**	3.13E+2(9.37E+1)	6.41E+0
	$T_{2}$	1.33E−8(3.41E−8)		1.94E−2(8.38E−2)	
5	$T_{1}$	1.85E+0(9.61E−1)	**−3.77E+1**	3.26E+0(6.39E−1)	3.77E+1
	$T_{2}$	8.56E+1(2.75E+1)		1.64E+2(6.83E+1)	
6	$T_{1}$	3.50E+0(8.06E−1)	**−1.95E+1**	3.21E+0(6.83E−1)	1.95E+1
	$T_{2}$	3.65E+0(1.19E+0)		9.77E+0(2.48E+0)	
7	$T_{1}$	8.48E+1(4.22E+1)	**−4.15E+1**	2.33E+2(1.66E+2)	4.15E+1
	$T_{2}$	8.27E+1(3.51E+1)		3.26E+2(9.38E+1)	
8	$T_{1}$	9.28E−3(8.61E−3)	**−2.25E+1**	8.57E−3(8.81E−3)	2.25E+1
	$T_{2}$	2.02E+1(3.39E+0)		3.11E+1(4.93E+0)	
9	$T_{1}$	9.50E+1(1.25E+2)	**−1.59E+1**	3.15E+2(1.04E+2)	1.59E+1
	$T_{2}$	1.62E+2(6.98E+2)		8.19E−1(1.73E+0)	
Mean		-	**−2.38E+1**	-	2.38E+1

**Table 5:** Comparison of SRPSMTO, MFPSO, SREMTO and MFEA in terms of the means, bracketed standard deviations and scores of the best achieved FEVs over 30 runs on nine MTO problems in test suite 1. The best results are shown in bold.

Problem	Task	SRPSMTO_V1		SRPSMTO_V2		MFPSO		SREMTO		MFEA	
		Mean(Std)	Score	Mean(Std)	Score	Mean(Std)	Score	Mean(Std)	Score	Mean(Std)	Score
1	$T_{1}$	3.45E−3(7.55E−3)	**−3.90E+1**	6.17E−3(8.16E−3)	−3.63E+1	9.34E−1(8.34E−2)	1.13E+2	1.09E−2(9.83E−3)	−3.49E+1	9.64E−2(2.33E−2)	−2.76E+0
	$T_{2}$	1.69E+1(3.33E+1)		2.86E+1(4.06E+1)		3.76E+2(2.95E+1)		3.33E+1(2.72E+1)		1.53E+2(5.65E+1)	
2	$T_{1}$	2.84E+0(6.62E−1)	**−4.40E+1**	3.35E+0(7.81E−1)	−3.16E+1	7.29E+0(8.78E−1)	1.05E+2	3.32E+0(8.88E−1)	−3.74E+1	4.57E+0(8.90E−1)	8.39E+0
	$T_{2}$	6.76E+1(2.67E+1)		9.15E+1(4.48E+1)		5.19E+2(6.70E+1)		5.92E+1(2.66E+1)		2.11E+2(6.44E+1)	
3	$T_{1}$	1.01E−2(9.24E−3)	**−6.22E+1**	1.29E−1(1.93E−1)	−6.18E+1	2.12E+1(4.19E−2)	8.08E+1	2.09E+1(4.07E−1)	3.03E+1	2.01E+1(7.57E−2)	1.29E+1
	$T_{2}$	7.15E−3(1.04E−2)		6.20E−1(1.37E+0)		1.42E+4(7.84E+2)		5.48E+3(6.45E+2)		2.81E+3(4.23E+2)	
4	$T_{1}$	2.83E+2(7.26E+1)	−3.44E+1	3.03E+2(9.57E+1)	−3.19E+1	8.49E+2(9.53E+1)	1.10E+2	2.49E+2(6.04E+1)	**−3.86E+1**	5.18E+2(8.69E+1)	−5.04E+0
	$T_{2}$	1.82E−7(4.40E−7)		1.33E−8(3.41E−8)		4.50E+3(7.31E+2)		1.09E−15(2.82E−15)		3.71E−1(8.38E−2)	
5	$T_{1}$	1.44E+0(9.40E−1)	**−3.56E+1**	1.85E+0(9.61E−1)	−2.96E+1	6.49E+0(9.36E−1)	1.04E+2	2.04E+0(9.33E−1)	−2.69E+1	3.05E+0(7.10E−1)	−1.20E+1
	$T_{2}$	9.79E+1(3.11E+1)		8.56E+1(2.75E+1)		9.94E+4(4.92E+4)		9.55E+1(3.03E+1)		2.26E+2(8.01E+1)	
6	$T_{1}$	3.19E+0(8.22E−1)	**−4.57E+1**	3.50E+0(8.06E−1)	−4.00E+1	1.14E+1(1.59E+0)	1.98E+1	3.58E+0(7.35E−1)	−4.07E+1	1.99E+1(9.18E−2)	1.07E+2
	$T_{2}$	2.66E+0(8.16E−1)		3.65E+0(1.19E+0)		9.26E+0(1.59E+0)		3.42E+0(9.00E−1)		2.05E+1(2.92E+0)	
7	$T_{1}$	8.36E+1(4.07E+1)	−3.26E+1	8.48E+1(4.22E+1)	−3.03E+1	3.34E+5(1.23E+5)	1.10E+2	8.91E+1(5.66E+1)	**−3.36E+1**	2.94E+2(2.31E+2)	−1.35E+1
	$T_{2}$	6.69E+1(6.15E+1)		8.27E+1(3.51E+1)		5.70E+2(1.12E+2)		6.02E+1(2.32E+1)		1.97E+2(6.51E+1)	
8	$T_{1}$	5.75E−3(8.13E−3)	−3.90E+1	9.28E−3(8.61E−3)	−2.92E+1	1.11E+0(5.06E−2)	9.45E+1	8.70E−3(1.00E−2)	**−4.07E+1**	9.60E−2(2.12E−2)	1.44E+1
	$T_{2}$	1.85E+1(3.31E+0)		2.02E+1(3.39E+0)		2.86E+1(1.40E+0)		1.82E+1(3.00E+0)		2.68E+1(3.15E+0)	
9	$T_{1}$	1.42E+2(8.54E+1)	−4.62E+1	9.50E+1(1.25E+2)	**−4.81E+1**	1.50E+3(2.34E+2)	1.10E+2	2.46E+2(4.85E+1)	−1.01E+1	5.61E+2(1.04E+2)	−5.32E+0
	$T_{2}$	4.07E+1(1.04E+2)		1.62E+2(6.98E+2)		1.35E+4(1.43E+3)		5.11E+3(6.18E+2)		2.98E+3(3.92E+2)	
Mean		-	**−4.21E+1**	-	−3.77E+1	-	9.41E+1	-	−2.59E+1	-	1.15E+1

**Table 6:** Comparison of SRPSMTO (SRPSMTO_V1 and SRPSMTO_V2), PSO, SREMTO and MFEA in terms of the means, bracketed standard deviations and scores of the best achieved FEVs over 30 runs on test suite 2. The best results are shown in bold.

Problem	Task	SRPSMTO_V1		SRPSMTO_V2		PSO		SREMTO		MFEA	
		Mean(Std)	Score	Mean(Std)	Score	Mean(Std)	Score	Mean(Std)	Score	Mean(Std)	Score
1	$T_{1}$	9.33E+1(4.99E+1)	−5.99E+1	1.00E+2(5.70E+1)	**−6.32E+1**	1.80E+2(9.40E+1)	1.31E+1	1.91E+2(1.12E+2)	4.83E+0	3.43E+2(9.37E+1)	1.05E+2
	$T_{2}$	8.79E+1(3.94E+1)		7.73E+1(3.89E+1)		1.85E+2(1.13E+2)		2.31E+2(1.55E+2)		2.87E+2(6.41E+1)	
	$T_{3}$	9.38E+1(4.30E+1)		9.34E+1(4.14E+1)		3.85E+2(8.77E+2)		1.37E+2(4.02E+1)		5.71E+2(7.65E+2)	
	$T_{4}$	8.87E+1(4.36E+1)		6.93E+1(3.05E+1)		2.11E+2(1.34E+2)		1.42E+2(5.67E+1)		3.38E+2(9.57E+1)	
	$T_{5}$	9.46E+1(6.11E+1)		9.65E+1(3.21E+1)		1.63E+2(6.05E+1)		1.77E+2(1.57E+2)		2.81E+2(4.57E+1)	
2	$T_{1}$	1.22E−6(9.73E−7)	**−4.83E+1**	6.87E−2(3.07E−1)	−4.73E+1	2.86E+0(7.85E−1)	−5.41E+0	1.31E+1(9.89E+0)	1.47E+2	1.62E−1(4.46E−2)	−4.59E+1
	$T_{2}$	3.82E−6(3.93E−6)		6.87E−2(3.07E−1)		3.30E+0(3.59E+0)		1.31E+1(9.87E+0)		1.55E−1(4.02E−2)	
	$T_{3}$	2.70E−6(2.49E−6)		6.87E−2(3.07E−1)		2.42E+0(5.34E−1)		1.31E+1(9.87E+0)		1.68E−1(3.21E−2)	
	$T_{4}$	2.36E−6(1.93E−6)		6.87E−2(3.07E−1)		2.98E+0(9.81E−1)		1.32E+1(9.90E+0)		1.74E−1(3.12E−2)	
	$T_{5}$	1.42E−6(1.39E−6)		6.87E−2(3.07E−1)		2.88E+0(9.97E−1)		1.31E+1(9.90E+0)		1.65E−1(4.01E−2)	
3	$T_{1}$	3.19E+2(8.46E+1)	−3.68E+1	3.23E+2(9.69E+1)	−2.57E+1	3.38E+2(9.18E+1)	−2.59E+1	2.79E+2(1.39E+2)	**−6.04E+1**	5.63E+2(1.25E+2)	1.49E+2
	$T_{2}$	3.04E+2(5.87E+1)		3.03E+2(9.61E+1)		3.14E+2(6.60E+1)		2.52E+2(6.17E+1)		6.10E+2(1.76E+2)	
	$T_{3}$	2.92E+2(8.88E+1)		3.08E+2(8.15E+1)		2.98E+2(7.58E+1)		2.82E+2(1.27E+2)		5.93E+2(1.42E+2)	
	$T_{4}$	3.04E+2(9.15E+1)		3.19E+2(9.22E+1)		3.04E+2(9.86E+1)		2.57E+2(9.59E+1)		5.95E+2(1.01E+2)	
	$T_{5}$	2.97E+2(6.06E+1)		3.43E+2(1.07E+2)		3.40E+2(8.04E+1)		2.65E+2(9.19E+1)		5.73E+2(1.06E+2)	
4	$T_{1}$	6.03E−3(8.50E−3)	**−5.13E+1**	5.66E−3(1.12E−2)	−5.03E+1	1.35E−2(1.08E−2)	−3.77E+1	1.19E−2(1.17E−2)	−4.78E+1	1.37E−1(3.29E−2)	1.87E+2
	$T_{2}$	9.36E−3(1.18E−2)		8.99E−3(9.76E−3)		2.37E−2(4.29E−2)		4.74E−3(7.55E−3)		1.26E−1(3.18E−2)	
	$T_{3}$	6.65E−3(8.61E−3)		4.80E−3(9.05E−3)		7.75E−3(8.27E−3)		7.48E−3(1.14E−2)		1.42E−1(3.18E−2)	
	$T_{4}$	4.93E−3(5.65E−3)		7.51E−3(9.42E−3)		1.57E−2(1.84E−2)		1.11E−2(1.11E−2)		1.37E−1(2.74E−2)	
	$T_{5}$	6.65E−3(7.50E−3)		9.47E−3(1.16E−2)		9.29E−3(9.91E−3)		8.05E−3(7.52E−3)		1.45E−1(2.60E−2)	
5	$T_{1}$	5.52E+0(1.49E+0)	1.19E+1	3.89E+0(1.10E+0)	−3.14E+1	5.27E+0(1.18E+0)	6.98E−1	8.74E+0(2.98E+0)	1.35E+2	1.34E+0(1.51E−1)	**−1.16E+2**
	$T_{2}$	5.50E+0(1.16E+0)		4.07E+0(1.36E+0)		5.30E+0(1.78E+0)		9.89E+0(3.92E+0)		1.35E+0(1.03E−1)	
	$T_{3}$	5.29E+0(1.66E+0)		4.55E+0(1.43E+0)		5.47E+0(1.68E+0)		1.06E+1(3.68E+0)		1.28E+0(1.21E−1)	
	$T_{4}$	5.57E+0(1.65E+0)		4.37E+0(1.32E+0)		4.66E+0(1.72E+0)		9.53E+0(3.96E+0)		1.34E+0(1.49E−1)	
	$T_{5}$	6.26E+0(1.62E+0)		4.05E+0(1.25E+0)		5.57E+0(1.42E+0)		1.02E+1(3.60E+0)		1.37E+0(1.35E−1)	
6	$T_{1}$	2.80E+2(3.67E+2)	−8.18E+1	6.53E+2(4.31E+2)	−6.07E+1	2.13E+2(1.83E+2)	**−8.51E+1**	4.24E+3(8.23E+2)	1.47E+2	3.15E+3(3.48E+2)	8.01E+1
	$T_{2}$	2.56E+2(3.26E+2)		6.33E+2(4.10E+2)		1.85E+2(1.48E+2)		4.36E+3(8.93E+2)		3.18E+3(4.30E+2)	
	$T_{3}$	2.90E+2(3.94E+2)		6.29E+2(3.90E+2)		2.16E+2(1.61E+2)		4.22E+3(8.25E+2)		3.08E+3(3.83E+2)	
	$T_{4}$	3.00E+2(3.77E+2)		6.91E+2(4.19E+2)		2.69E+2(2.24E+2)		4.41E+3(8.17E+2)		2.99E+3(3.62E+2)	
	$T_{5}$	2.86E+2(3.72E+2)		6.45E+2(4.00E+2)		2.36E+2(1.78E+2)		4.14E+3(7.82E+2)		3.11E+3(4.38E+2)	
Mean		-	-4.44E+1	-	**−4.64E+1**	-	-2.34E+1	-	5.43E+1	-	5.98E+1

**Table 7:** Achieved best FEVs (the mean, the bracketed standard deviations and the scores) by the SRPSMTO using different population size (*n*) settings. The better results are shown in bold.

Problem	Task	*n* = 20		*n* = 60		*n* = 100		*n* = 140		*n* = 180	
		Mean(Std)	Score	Mean(Std)	Score	Mean(Std)	Score	Mean(Std)	Score	Mean(Std)	Score
1	$T_{1}$	5.15E−1(2.15E−1)	1.02E+2	8.74E−3(1.02E−2)	−2.33E+1	3.45E−3(7.55E−3)	**−3.19E+1**	8.05E−3(1.02E−2)	−2.37E+1	7.93E−3(9.37E−3)	−2.28E+1
	$T_{2}$	1.67E+2(4.87E+1)		3.53E+1(3.94E+1)		1.69E+1(3.33E+1)		3.46E+1(3.82E+1)		3.67E+1(4.81E+1)	
2	$T_{1}$	6.42E+0(1.21E+0)	9.76E+1	3.56E+0(1.04E+0)	−1.97E+0	2.84E+0(6.62E−1)	−2.50E+1	2.48E+0(5.95E−1)	−3.20E+1	2.34E+0(4.39E−1)	**−3.86E+1**
	$T_{2}$	2.58E+2(1.08E+2)		1.01E+2(5.06E+1)		6.76E+1(2.67E+1)		6.53E+1(2.69E+1)		5.26E+1(1.61E+1)	
3	$T_{1}$	2.07E−1(2.76E−1)	−2.13E+0	2.80E−2(2.80E−2)	−2.78E+1	1.01E−2(9.24E−3)	**−2.97E+1**	3.35E−1(4.10E−1)	2.17E+1	4.36E−1(4.03E−1)	3.80E+1
	$T_{2}$	1.19E+0(2.50E+0)		4.28E−2(7.15E−2)		7.15E−3(1.04E−2)		2.70E+0(5.44E+0)		3.57E+0(4.98E+0)	
4	$T_{1}$	6.07E+2(1.27E+2)	8.03E+1	3.33E+2(8.05E+1)	−1.08E+1	2.83E+2(7.26E+1)	−2.03E+1	2.75E+2(7.82E+1)	−2.17E+1	2.45E+2(7.89E+1)	**−2.75E+1**
	$T_{2}$	5.79E+1(8.40E+1)		4.93E−5(1.42E−4)		1.82E−7(4.40E−7)		1.05E−5(5.60E−5)		3.50E−5(8.50E−5)	
5	$T_{1}$	4.02E+0(6.84E−1)	8.53E+1	2.00E+0(7.53E−1)	−1.17E+1	1.44E+0(9.40E−1)	−2.52E+1	1.55E+0(9.08E−1)	−2.28E+1	1.43E+0(8.59E−1)	**−2.55E+1**
	$T_{2}$	2.66E+3(2.55E+3)		1.21E+2(4.53E+1)		9.79E+1(3.11E+1)		9.50E+1(2.96E+1)		9.48E+1(2.36E+1)	
6	$T_{1}$	8.70E+0(1.68E+0)	1.06E+2	3.98E+0(8.47E−1)	−6.57E+0	3.19E+0(8.22E−1)	−2.88E+1	3.04E+0(7.78E−1)	−3.02E+1	2.58E+0(6.52E−1)	**−4.07E+1**
	$T_{2}$	8.25E+0(1.55E+0)		3.70E+0(1.07E+0)		2.66E+0(8.16E−1)		2.70E+0(1.02E+0)		2.30E+0(7.21E−1)	
7	$T_{1}$	2.49E+3(2.46E+3)	8.33E+1	1.33E+2(5.48E+1)	−1.51E+1	8.36E+1(4.07E+1)	**−2.39E+1**	9.26E+1(4.36E+1)	−2.11E+1	9.75E+1(5.58E+1)	−2.32E+1
	$T_{2}$	2.38E+2(9.24E+1)		9.00E+1(3.76E+1)		6.69E+1(6.15E+1)		7.47E+1(5.67E+1)		6.80E+1(4.18E+1)	
8	$T_{1}$	6.47E−1(2.86E−1)	9.39E+1	9.16E−3(8.62E−3)	−1.23E+1	5.75E−3(8.13E−3)	−2.07E+1	5.99E−3(9.00E−3)	**−3.35E+1**	7.15E−3(8.70E−3)	−2.73E+1
	$T_{2}$	2.48E+1(2.50E+0)		1.96E+1(2.68E+0)		1.85E+1(3.31E+0)		1.68E+1(2.58E+0)		1.76E+1(3.12E+0)	
9	$T_{1}$	3.13E+2(1.25E+2)	5.70E+1	2.00E+2(8.92E+1)	−2.74E+0	1.42E+2(8.54E+1)	−1.37E+1	1.38E+2(8.08E+1)	−1.67E+1	1.24E+2(9.64E+1)	**−2.38E+1**
	$T_{2}$	2.13E+2(3.48E+2)		1.78E+1(6.53E+1)		4.07E+1(1.04E+2)		2.90E+1(1.28E+2)		6.61E+0(1.02E+1)	
Mean		-	7.81E+1	-	−1.25E+1	-	**−2.44E+1**	-	−2.00E+1	-	−2.13E+1

## Data Availability

All data generated or analyzed during this study are included in this published article.

## Abbreviations

The following abbreviations are used in this manuscript:

MTO	Multi-task Optimization
EMTO	Evolutionary Multi-task Optimization
SREMTO	Self-regulated Evolutionary Multi-task Optimization
PSO	Particle Swarm Optimization
MFEA	Multifactorial Evolutionary Algorithm
MFPSO	Multifactorial Particle Swarm Optimization
MT-CPSO	Multitasking Coevolutionary Particle Swarm Optimization
MTMSO	Multitasking Multiswarm Optimization
